# Retinitis after haematopoietic stem cell transplantation with multiple intraocular viral infections (cytomegalovirus, Epstein‒Barr virus and herpes simplex virus)- a case report

**DOI:** 10.1186/s12886-024-03300-4

**Published:** 2024-01-24

**Authors:** Mengyun Liu, Hengqian He, Juntao Zhang, Shuanghua Xin, Qinkang Lu, Lifang Zhang, Weina Ren

**Affiliations:** Department of Ophthalmology, The Affiliated People’s Hospital of Ningbo University, The Eye Hospital of Wenzhou Medical University (Ningbo Branch), 315040 Ningbo, People’s Republic Of China

**Keywords:** Retinitis, Hematopoietic stem cell transplantation, CMV, EBV, HSV

## Abstract

**Background:**

To report a case of retinitis with multiple intraocular viral infections after second haematopoietic stem cell transplantation.

**Case presentation:**

A 39-year-old female patient developed retinitis after a second haematopoietic stem cell transplant. Right eye was tested for three viral infections– cytomegalovirus, Epstein‒Barr virus and herpes simplex virus, while left was infected with cytomegalovirus. The patient was subsequently treated with vitreous cavity ganciclovir injections, and 1 week later both eyes tested negative for aqueous humour viruses.

**Discussion and conclusion:**

CMV, EBV and HSV belong to the herpes virus family. They are all commonly observed in the body and represent opportunity infectious viruses. The retinitis they cause have different characteristics. But simultaneous infection of the eye by multiple viruses is quite rare. In this case, three viruses were detected in the patient’s eye, but whether the retina was caused by all three viruses at the same time could not be determined. A satisfactory outcome was achieved after treatment with vitreous cavity ganciclovir injection.

## Background

Acute lymphocytic leukaemia is a blood malignancy caused by malignant proliferation of lymphocytes in the blood and bone marrow [1]. The manifestations of acute lymphoblastic leukaemia is vary. Chemotherapy and haematopoietic stem cell transplantation(HSCT) are the common and very effective treatments [2]. Despite the effectiveness of haematopoietic stem cell transplantation in the treatment of acute lymphoblastic leukaemia, it continues to present many challenges. The origin of these problems is the immunosuppression that occurs during transplantation [3].

Opportunistic infections with intraocular viruses are often secondary conditions in immunosuppressed patients-such as individuals with acquired immunodeficiency syndrome(AIDS) and posthaematopoietic stem cell transplantation patients [4]. Cytomegalovirus(CMV) retinitis is the most common complication in these patients [5, 6]. CMV is a double-stranded DNA virus that is a member of the herpesvirus family [7]. Herpes viruses are usually divided into eight species. In addition to CMV, herpes simplex virus(HSV), varicella-zoster virus(VZV), and Epstein‒Barr virus(EBV) are also herpes viruses [6]. Retinitis caused by viruses other than CMV is uncommon in immunosuppressed patients, and mixed infections with multiple viruses are even rarer.

Within our limited knowledge, we have not seen reports of retinitis patients after haematopoietic stem cell transplantation who were coinfected by kinds of herpes viruses. In this case report, we describe a haematopoietic stem cell transplantation recipient who presented with retinitis caused by cytomegalovirus, Epstein‒Barr virus and herpes simplex virus.

## Case presentation

**Fig. 1:**
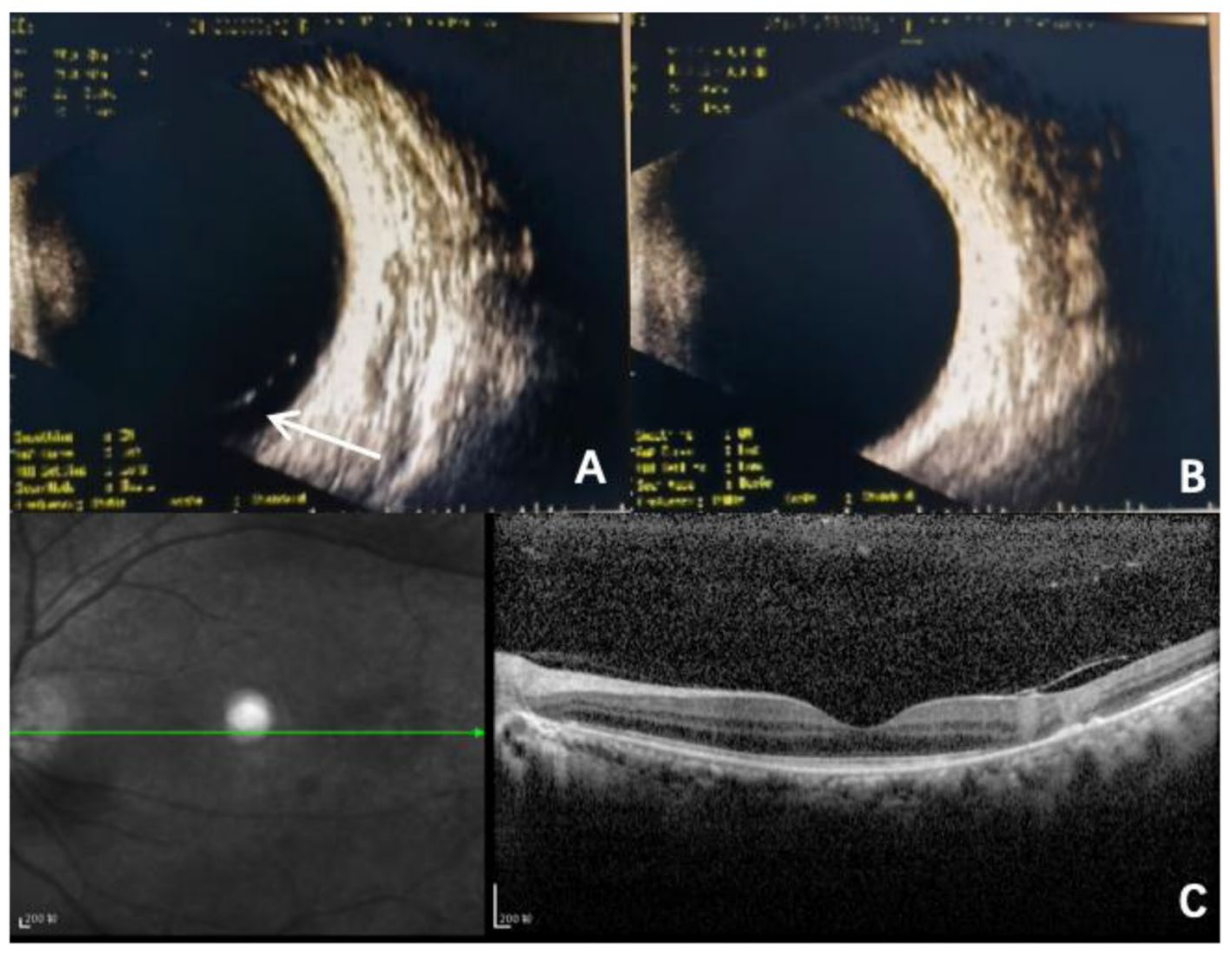
**(A)** Ultrasound of the eye indicates significant clouding of the vitreous humour below the right eye (The white arrow). **(B)** There is no significant abnormality in the ultrasound of the left eye. **(C)** OCT suggested enhanced signal in part of the retina of the right eye, with partial absence of the photoreceptor cell layer

**Fig. 2:**
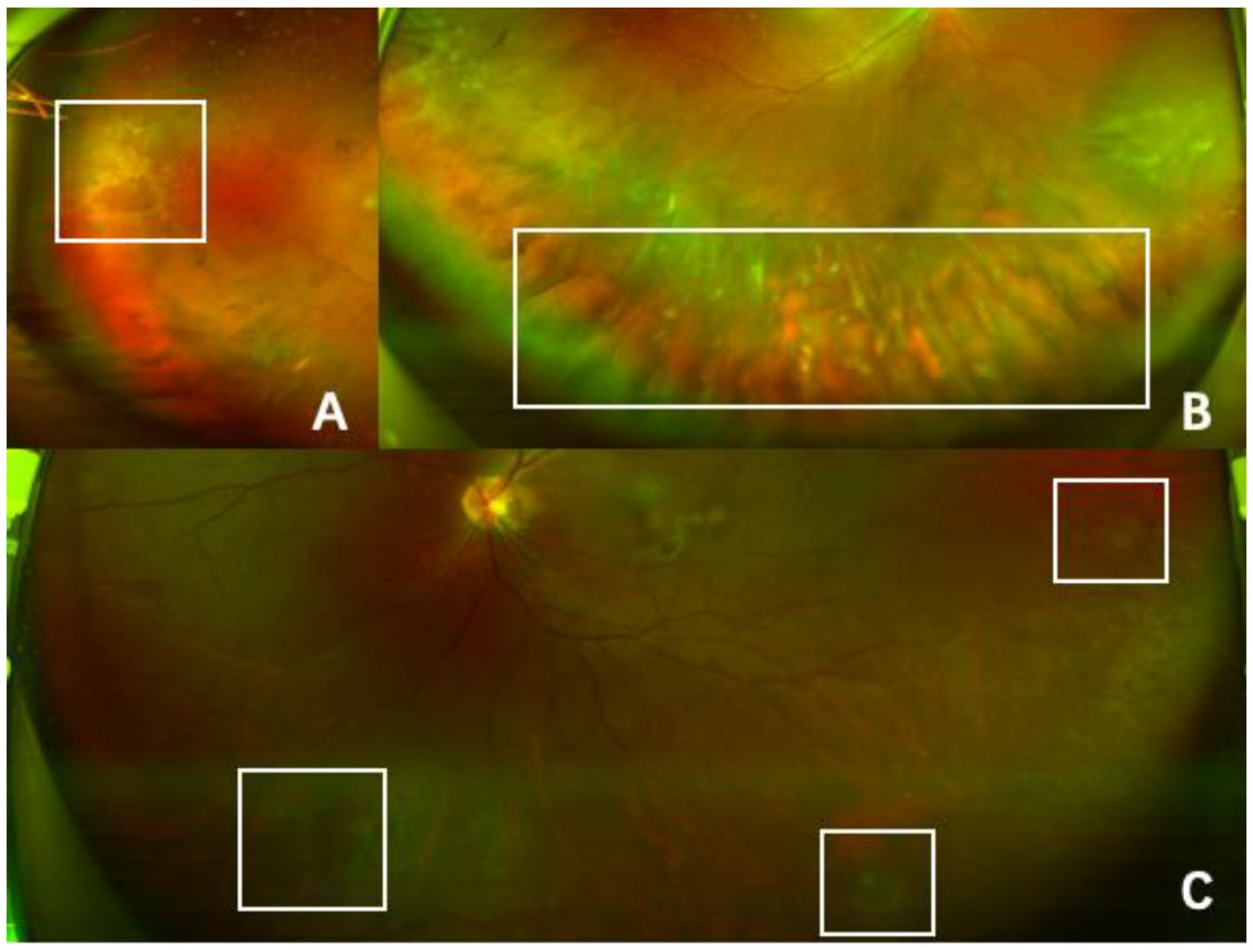
**(A)** A yellowish-white exudate with hemorrhage was seen in the peripheral part of the retina in the direction of 9 o’clock (White rectangle). **(B)** Inferior vitreous opacity was evident, and the lower portion of the retina could not be seen (White rectangle). **(C)** Multiple small degenerations were observed in the retina of the left eye (White rectangle)

In April 2023, a 39-year-old Chinese woman presented to our hospital complaining of a black shadow in front of her right eye for 5 days. This patient had been diagnosed with acute lymphoblastic leukaemia in 2017 and received an autologous HSCT in 2018. Unfortunately, she suffered a relapse of leukaemia and underwent an allogeneic HSCT in October 2022. The patient developed ocular symptoms at 6 months after receiving the transplant. After the consultation, the patient received a series of tests including visual acuity, slit lamp, noncontact tonometer, ocular ultrasound, macular optical coherence tomography, and Optos fundus photography. The patient’s best corrected visual acuity was 20/20 in both eyes using the Snellen visual acuity chart. The intraocular pressure was within the normal range. Slit lamp examination revealed that the anterior segmental structures of both eyes were normal. Ultrasound of the eye suggested flocculent vitreous clouding in the lower part of the right eye (Fig. 1). Partial signal enhancement in the retina of the left eye was found, and part of the photoreceptor cell layer was missing on optical coherence tomography(OCT) (Fig. 1). Fundus photography revealed a yellowish-white exudate with haemorrhage in the peripheral retina of the right eye and a faint haemorrhage in the lower retina of the left eye (Fig. 2). Combining the medical history and relevant ophthalmic examination, we highly suspected that the patient had developed cytomegalovirus retinitis.

**Fig. 3:**
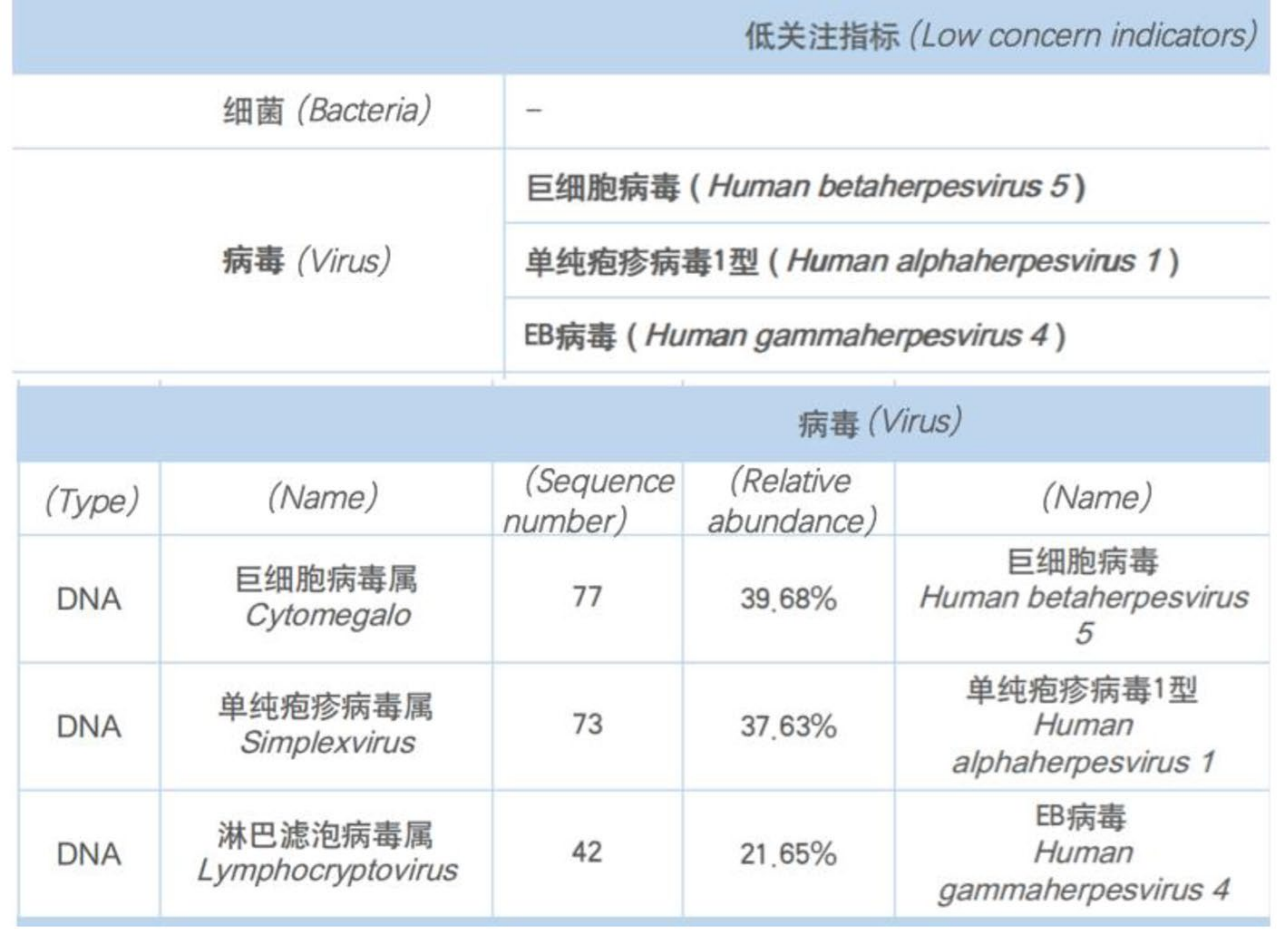
No bacteria were detected in the aqueous humour, while three viruses, including CMV, HSV and EBV, were identified. Their relative abundances were respectively 39.68%, 37.63% and 21.65%

**Fig. 4:**
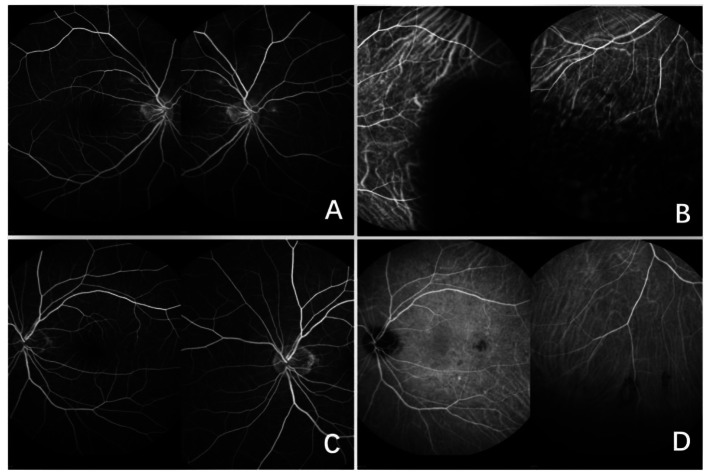
**(A)** A small amount of fluorescence leakage is seen in the retinal FFA of the right eye. **(B)** ICGA shows extensive fluorescence obscuration in the subnasal and inferior choroid of the right eye. **(C)** No significant abnormalities were seen in the FFA of the left eye. **(D)** Multiple small patches of obscured fluorescence were seen in the ICGA of the left eye

Therefore, we performed anterior atrial puncture and extracted aqueous humour from the patient for viral testing on 23, April. We further refined FFA + ICGA on April 25. Surprisingly, utilizing broad-spectrum high-throughput sequencing, it was found that the aqueous humour of her right eye tested positive for three viral DNAs-CMV, EBV, and HSV (Fig. 3). Internal markings and no template control were added to rule out a false detection of both herpes viruses during the high-throughput sequencing. The left eye was positive for CMV only. The FFA + ICGA findings indicated a small amount of retinal exudate in the patient’s right eye, and a large area of obscured fluorescence in the subnasal and inferior choroid (Fig. 4). The patient was then treated with bilateral vitreous cavity ganciclovir injections. Viral DNA was retested one week later and no virus was detected in the aqueous humour of either eye by quantitative polymerase chain reaction(QPCR). The test was negative for viral DNA. OCT and fundus photography indicated no significant changes in the fundus. The patients underwent weekly serum cytomegalovirus and EBV testing after the second stem cell transplant. She was cytomegalovirus positive only between November 15, 2022 and January 14, 2023, with viral copy numbers ranging from 1.02 × 10^2^ to 2.37 × 10^4^ during this period. Each test was negative for EBV. The patient was treated with systemic antiviral therapy in the hematology department since February, 2023. As of May 22, 2023, her fundal lesions all remained stable.

## Discussion and conclusion

Cytomegalovirus infections are common in posttransplant patients, especially in the graft-versus-host disease (GVHD) and immunosuppressed patients. Even serum positivity for CMV is as high as 92.2% [8]. In half of the patients, prior to the onset of ocular symptoms, patients develop other systemic CMV infection signs, most commonly including gastrointestinal disorders and interstitial pneumonitis. It has been otherwise reported in the literature that CMV retinitis is usually diagnosed within 106–365 days after transplantation, with a median of 251 days [9]. This timeframe appears to be the window of high incidence of CMV retinitis. The patient in this case was diagnosed approximately six months after transplantation. However, a thorough examination of the patient’s internal medicine findings did not reveal the presence of chronic GVHD in the individual, and in contrast to typical CMV retinitis, three distinct viruses were detected in her ocular region.

CMV, HSV and EBV infections are opportunistic infections [10]. They are fairly common in the body, and EBV is lifelong in 90% of people [11]. Moreover, these viruses have been implicated in the aetiology of ARN [12]. The evidence of CMV and EBV causing ARN is not robust enough [13]. Retinitis caused by various viruses in the herpesvirus family has its own characteristics; patients with retinitis caused by HSV are usually immunocompetent, while CMV is more common in immunodeficient individuals [14]. The most critical feature of ARN is necrotizing retinitis involving the peripheral retina [13]. In combination with a positive PCR for HSV or VZV in the aqueous humour or vitreous specimen and a characteristic clinical picture including– (a) ring or fusion retinitis, and (b) retinal vascular sheaths and/or occlusions, and (c) vitritis of more than mild severity, the diagnosis of ARN is confirmed [13]. PCR is the best way to detect HSV. ARN progresses quickly, and treatment typically does not need to be postponed until the return of PCR results. Indeed, the earlier the treatment, the better the prognosis for vision [15, 16]. EBV retinitis is very rare. EBV retinitis tends to result in the chronic uveitis which is characterized by vitreous strands and snowball-shaped lower portions, optic disc oedema, massive retinitis and retinal haemorrhages, and diffuse macular oedema [17]. And EBV tends to cause severe retinitis, which is difficult to treat with various treatments [17, 18]. While CMV retinitis is quite common in immunodeficient patients. The extent of retinal damage in our patient was mild and no evidence of ARN was detected. Despite the three viruses identified in the patient’s aqueous humour, we still considered CMV to be the cause of the retinitis. Although these three viruses are often detected in inflammatory ophthalmologic lesions [19], coinfection with multiple herpes viruses is rare due to their different infection characteristics of these viruses.

The patient’s aqueous humour was positive for CMV, HSV and EBV DNA, but her blood was negative for CMV and EBV at the time of ocular symptoms. The patient’s blood has never been positive for EBV since her initiation of EBV testing. Although EBV virus is not detected in the blood, the virus is likely to be latent in the body. It has been reported in the literature that EBV virus can be detected in about 20% of cadaveric eye tissues [20]. This may have contributed to the phenomenon. The literature has previously reported a number of patients with dual viral infections [21–23]. Such cases are extremely rare. We believe that the eye is a relatively independent organ due to the presence of multiple barriers, resulting in viruses in the blood being relatively independent of viruses in the eye. Therefore, the situation of the viruses in the blood does not reflect the situation of the intraocular viruses, which is likely the reason for the different results of aqueous humour and blood virus tests. We have never been clear as to why three viruses were detected in the patient’s aqueous humour. Indeed, we have not seen any similar reports in the past. Thus, we hypothesize that these three viruses coincidentally entered the eye successively within a short period of time while the patient was in an immunocompromised state, and at the same time the viruses escaped the surveillance of immune cells in the eye but could not enter the bloodstream due to the presence of multiple blood-eye barriers in the eye. Eventually, the three viruses simultaneously infected in the eye. Of course, although three viruses were detected in the patient’s eye, this does not mean that all three viruses caused retinitis. In particular, whether EBV can cause retinitis in immunodeficient patients remains highly controversial. The vitreous cavity injection of ganciclovir treatment was effective essentially ruling out the possibility of EBV retinitis. Therefore, the final and most likely diagnosis for this patient remains CMV retinitis. However, the evidence of simultaneous intraocular infection by all three viruses is clear. As the concomitant detection of all three viruses in the same patient’s eye is still quite rare, this case has certain research value.

Fortunately, all three viruses were disappeared after vitreous cavity injections of ganciclovir. With only one vitreous cavity injection, the patient’s aqueous humour screen was negative for virual infection. The fundal condition of the patient also remained stable for the time being.

Through this case, we were still able to recognize that it is still quite necessary to perform aqueous humour testing in such immunodeficient patients once they present with ocular symptoms. This is because (1) blood virus testing is hardly a true reflection of the viral situation in the eye, and (2) CMV infection is most common in this type of patient, but other viruses are also present with the possibility of infection, especially those usually thought to be present in immunocompetent individuals.

Although we currently do not know why patients are infected with these viruses at the same time, we hope to conduct further research to avoid multiple infections as much as possible and to be able to protect the vision of these unfortunate patients to the greatest extent achievable.

## Data Availability

Supporting data for the results of this study are available from the authors upon request. The e-mail address is 21718142@zju.edu.cn.

## Appendix

**Table:** QPCR probes and primers

Virus	Forward primer	Reverse primer	Probe
CMV	CCAACAGGATCATCGACCTCA	GAATCTCCTCGAGAATATGCTTGATTT	ACCCGGTGTTCAACAAGCTCC
VZV	TTGAACTGGATCTGTAGAGCAAC	CCTCTAAATCACATAACAGGTCACT	CAGACCGCGATGTAAGCCGAA
HSV	GCATCGCGGTGGTCTTCA	GAGTAGCGGTGGCCGAA	TCAAGGCCACCATGTACTACAAAGAC
EBV	CCCGGATAGACCTGGATGAC	CCCAAATTAAATAGTGATGCCAAAGAC	ATGCGTGTGATGATGACCTACCT

## Abbreviations

AIDS: Acquired immunodeficiency syndrome
CMV: Cytomegalovirus
HSV: Herpes simplex virus. VZV:varicella-zoster virus
EBV: Epstein-Barr virus
HSCT: Hematopoietic stem cell transplant
OCT: Optical coherence tomography
FFA: Fundus fluorescein angiography
ICGA: Indocyanine green angiography
QPCR: Quantitative Polymerase Chain Reaction
